# Gender and sex bias in prevention and clinical treatment of women’s chronic pain: hypotheses of a curriculum development

**DOI:** 10.3389/fmed.2023.1189126

**Published:** 2023-07-25

**Authors:** Chiara Moretti, Enrico De Luca, Clelia D’Apice, Giovanna Artioli, Leopoldo Sarli, Antonio Bonacaro

**Affiliations:** ^1^Department of Medicine and Surgery, University of Parma, Parma, Italy; ^2^Faculty of Health and Life Sciences Exeter University, Exeter, United Kingdom; ^3^Department of Nursing, University of Suffolk, Ipswich, United Kingdom

**Keywords:** chronic pain, women, gender bias, education, gender medicine

## Abstract

This discursive paper focuses on undergraduate medical education’s role in tackling gender bias in clinical practice, specifically preventing and managing from a non-biomedical perspective chronic pain in women. A preliminary web search of medical schools’ curricula was performed to identify programs content related to gender bias in pain management. The web search included 10 universities’ websites selected from the top 10 rankings QS Universities Rankings 2022 for medical schools. Additionally, a questionnaire was sent to all deans of the selected academic institutions to explore the curriculum content further. The web search, and the lack of response from the deans, highlighted that relevant curriculum components on gender bias and chronic pain needed to be implemented. Therefore, this paper introduces an innovative curriculum development approach designed by the multi-professional research team to be implemented in medical school programs. This novel educational strategy could also cross-contaminate other healthcare practitioners’ university programs and, thus, stimulate an interprofessional debate into fostering inclusiveness and equal opportunities in health.

## 1. Introduction

Medical education has been concerned with providing predominantly undergraduate students with the knowledge of the biophysical determinant of illnesses of the human body (1). With this discursive paper, an interprofessional team of academics and researchers from anthropology, nursing, education, medical science, and public health aimed to design a curriculum development intervention for medical education on psychosocial aspects of health, specifically chronic pain and gender.

Western medicine is transitioning from a clinical and biological model to a new one developed around the more exhaustive definition of health as per the WHO definition that says “...a state of complete well-being, mental and not the mere absence of the state of disease or infirmity.”

The so-called patient-centered medicine aims (2, 3) at placing the person at the center of care and at defining *disease* not only as a mere organic/physiological dysfunction of the organism (disease) but as a complex phenomenon experienced, both individually (illness) and socially (sickness) (4, 5). Nevertheless, Western medicine remains firmly rooted in biological determinism and the need to rely on data and evidence to offer answers using the most advanced technology. This biological determinism not only shapes medical and operational practices in healthcare settings but also strongly characterizes curricula and university programs attended by healthcare students and professionals. The limits of an exclusively “technical” approach with scant attention to the person emerge clearly when biomedicine is confronted with chronic pain, especially when the patient is female. Chronic pain is an enigma for evidence-based medicine because it is invisible. It cannot be traced objectively and visually in the organism, it cannot be examined by the “medical gaze” (6), and thus it escapes from empirical supervision. However, on the other hand, pain is also an experience (7): it is not a mere physiological sensation but, instead, is the result of an elaboration process of this sensation, where emotional, cognitive, individual, social, cultural, and environmental factors play a central role. In this sense, pain takes shape in the peculiarity of individual biographies, also manifesting itself as a “total” (8), having a multidimensional impact and affecting several aspects of life (psychic, social, relational, etc.). Therefore, pain management should be based on a holistic and personalized care approach. This approach contributes to defining *pain* as a multidimensional experience shaped by several elements. Among these elements are sex and gender differences that influence health and disease, particularly the processes of pain chronicization (9–12).

Chronic pain primarily affects the female population (13–16). However, most studies investigating pain mechanisms are mainly conducted on the male population (17–20), while physiological mechanisms underlying women’s pain and gender/sex differences are yet to be explored in-depth. The same is true for pharmacology (21, 22): most drugs are tested on the 70-kilo ideal male type, and the recommended dosages refer to this standard, even if women and men have different abilities to metabolize the active ingredients (23).

In literature, and especially in gender medicine studies, it has been repeatedly highlighted how doctors’ scant consideration and preparation for women’s health and female patients’ pain may be responsible for gender bias^1^ in care settings (23–27). Gender stereotypes influence biomedical ways of analyzing, interpreting, and treating female pain. For instance, several studies have shown that female pain is often underestimated since health professionals tend to frame it as a phenomenon amplified by behavioral and attitudinal traits that are considered to be “typically” females–such as amplified emotionality, psychological vulnerability and dramatization–which would lead them to less tolerate pain and manifest it exaggeratedly (24, 28–30). This leads professionals to be more inclined to interpret the symptoms reported by the male population as organic and those reported by the female population as psychosomatic, underestimating them (31, 32). The invisibility of pain and the difficulty in identifying a specific pathology that causes chronic pain also lead to the stigmatization of women suffering from complex chronic pain syndromes, as is well shown in the literature (29, 30, 33–35). Many women with chronic pain report that they were not believed and that their pain was not treated (29, 33).

The gender biases within a clinician-person relationship can result in a lack of equity and access to care. Literature (24, 36) is rich in examples of such biases. Compared to men, women receive less intensive and effective pain care (37–42). Women are less likely to be prescribed analgesics and opiates (37, 43) and more likely to receive antidepressant prescriptions than men (38). Furthermore, when male and female patients express the same type of pain, female patients are more likely to be referred to psychotherapy, while men receive pharmacological medications (29, 42, 44, 45). Psychologizing women’s chronic pain not only leads to an underestimation of a massive health problem but also has a negative impact on their illness experience; many women, in fact, state that they feel abandoned by health professionals, family members or partners, friends, and colleagues (29, 46).

Physicians’ gender stereotypes are responsible for inequalities in medicine and clinical practice. Research must examine how these stereotypes generate care disparities and influence patient–professional interactions. Therefore, to reduce gender bias, the awareness of values and attitudes toward gender must increase within the medical society. A possible approach is including gender theory and discussions in medical school curricula. However, a growing body of literature shows how gender medicine, specifically women’s health, is almost neglected in the medical schools’ curricula (47, 48). In addition, 70% of postgraduate physicians in training indicated that gender medicine concepts are never or sporadically discussed in their training program (49).

Because sex and gender are health determinants, incorporating these contents into medical curricula could promote a more comprehensive patient-centered approach. Research should focus on understanding how basic pain mechanisms may differ in the two sexes and the diverse ways in which gender differences currently influence diagnostic and treatment decisions. In addition, optimal pain management requires that clinicians understand and examine their gender stereotypes and be prepared to evaluate whether these stereotypes result in less-than-optimal pain management for specific individuals. This paper aims to analyze whether courses centered on gender medicine and, in particular, on female pain are provided in medical schools’ curricula. The paper aims to design a medical school’s curriculum innovation intervention for tackling gender-based health inequalities and fostering a biopsychosocial model for preventing and treating pain.

## 2. Method

The project implied several explorative steps allowing the research team to reflect on and design a suitable new course aiming at tackling gender bias in pain management in medical students.

The first step consisted of browsing the available web content of 10 worldwide top universities and medical schools (50) programs. Specifically, the web search sought any humanistic or social sciences lectures, training, or awareness courses within the programs selected. This first step was necessary as it is highly probable that interventions or training proposals aimed at raising pain/gender awareness are included in program modules with a humanistic and relational focus. In fact, training medical students to orient their clinical lenses to observe pain as a complex and gendered phenomenon is directly linked with implementing their communication, psycho, and social skills (51, 52). We directed our attention to the manifest content of medical school websites as a retrospective process to help us answer our initial question. Therefore, we explored 10 of the most important worldwide medical schools’ curricula and the available content to uncover to what extent medical curricula may contribute to tackling gender-based health inequalities and promote a non-biomedical approach to managing pain in women.

The second step of the project aimed at collecting more information on the university’s websites mentioned above on the humanistic contents of their programs. A questionnaire was emailed to the Deans for students’ teaching and learning experience and heads of departments of each of the 10 involved medical schools. The research team designed the three open-ended questions to investigate education leaders’ awareness or possibility of foreseeing training on the project topic (Appendix 1). Three separate reminders were sent to elicit a response and maximize participation.

The last step consisted of the course design phase, considering the previous steps’ findings. Therefore, the research team designed an innovative educational program in medical education that combines authors’ different points of view and respective disciplines background.

The study took place under local regulations, and no ethical approval was sought from the university ethics committee. Therefore, no personal and sensitive data were expected to be collected according to the developed questionnaire. Moreover, the invited Deans/heads of the Department have yet to respond to the questionnaire; thus, only literature review data are presented in this article.

## 3. Results

The research team selected medical schools among the top 10 universities per several continents (Europe, Asia, America, and Canada) according to the Top 10 ranking QS Universities Rankings 2022 (Appendix 2). The team selected three universities per continent, plus one for the United Kingdom. Each university website was searched for downloadable programs and curriculum content. Therefore, the search aimed to detect humanities, social science, anthropology, psychology, sociology, and similar fields elements incorporated in the selected curricula. In addition, any element or suggestion about person-centered care, medical humanities, model of care, communication, and interprofessional approaches was also considered. Universities with these characteristics and access were included. Finally, the accessible university programs and curriculum were analyzed.

The general perception of the programs analyzed was limited space for humanistic content. The number of hours dedicated to these lectures is generally minimal compared to the number of hours devoted to formative sciences and clinical skills. However, this final material selection brought the team’s attention to several crucial aspects. Although supported and preannounced to have a humanistic approach to health, some universities only possess some teaching with this content in the actual program. For example, one university describes its approach to medicine as holistic, but this concept needs to be explained or expanded within the program.

However, the same medical school proposed a course in psychological medicine in the third year. An interesting aspect is represented by an Asian approach for first-year students with a module called a *journey to understand myself, society, human beings and human life*. Nevertheless, the contents of this very promising module are not accessible, but they give an idea of a more person-centered model. A North American university teaches the “narrative medicine” approach within foundational seminars across all the programs.

Interesting to notice that some academic contexts put the study of communication, medical humanities, or psychology in the first 2 years. At the same time, others dedicate seminars on the same topics in the second part of their pathway when they are more senior. The Canadian model (CANmeds) adopted by two selected universities (Europe and Toronto) is worth mentioning. CANmeds consists of an integrated model of care developed by and for physicians. According to this model, the students approach medicine from the first year in an integrated way, combining clinical skills with aspects of medicine more concerned with relationships and community health.

The response to the second step of our inquiry into medical education was very scarce. The educational leaders have yet to respond to emails containing the brief questionnaire. Therefore, the research team decided to expand the search for references further in postgraduate education courses or training in the medical and health profession. From this last purposive review, the team found specific courses for the medical profession (sometimes open to interprofessionality) that are more focused on gender medicine and pain management. This last fact highlights that, at this moment, the education on topics so sensitive and deep into the human experience of illness is relegated to the ones who are probably already interested in it or looking for further specialization. Among the different courses, Harvard University, with a master’s in public health, designed a course in Gender, Women and Health. In comparison, the University of Aberdeen has a course on Women’s Health in a Global Setting, open to all health professions.

## 4. Discussion

The findings from this last search inspired the research team to enrich their course proposal, investing in a longitudinal pathway throughout the medical degree.

Therefore, this group proposes a foundational course that should become integral to the medical training curriculum. The course should last 4 years, starting from the first year of studies, with consequential modules that deepen, each time, different topics. The authors propose a four-year course considering the average length of medical education programs. In addition, the proposed workshops embedded in each year of study will ensure proportionate integration to student’s clinical practical experience; therefore, this approach will support the student’s progression in a reflective and lifelong learning perspective (53).

The course will consist of a mix of teaching and learning strategies. In addition, the course will provide for each year an analysis of specific initial needs (teachers will also use that to reorient the program) and an assessment of learning, educational impact, and satisfaction at the end of each module. The program is detailed in the table here below.

**Table 1:** 

Course title: Health, gender, and chronic pain	
1st year–gender, medicine, and health	
Theme	Health and gender
Contents	I Module (1) Sex as health determinants (sex differences in organ and body systems) (2) Sex differences in effect and side effects of pharmaceuticals (3) Incorporating gender analysis into health research and interventions. II Module (1) Gender differences and their impact on pathogenesis, diagnosis, prevention, and medical care (2) Gender distribution of diseases in the population (3) The role of gender in shaping health inequities and how gender health inequalities affect health research and interventions.
Objectives	Develop skills in gender medicine
Teaching modalities	Autobiography Interactive lesson Case discussion Use of reflective diaries in clinical placement Reflective writing Final evaluation
Hours	20 h (frontal lectures and facilitated team-work)
2nd year–pain as a multidimensional experience	
Theme	The multiple aspects of pain
Contents	I Module The physiological basis of pain II Module The social/cultural aspects in pain III Module The psychological aspects in pain
Objectives	Understanding and deepening the multidimensionality of the pain symptoms
Teaching modalities	Initial autobiography Case discussion Interactive lesson Testimonials and reflections on the experience Use of reflective diaries in clinical placement Reflective writing Final evaluation
Hours	20 h
3rd year–gender medicine and female chronic pain	
Theme	Sex and gender differences in pain and pain management
Contents	I Module (1) Biologic factors may account for sex differences in pain (2) Psychosocial factors may account for sex/gender differences in pain sensitivity II Module (1) Medicine, pain, and gender bias (2) Pain, chronic pain, and women III Module (1) Reflect on one’s own gender stereotypes and their impact on clinical practice (2) Medical practice and self-reflexivity
Objectives	To Deepen the characteristics of sex and gender related to pain and chronic pain To reflect on gender biases in pain and pain management and develop skills to trace them
Teaching modalities	Initial autobiography Case discussion Interactive lesson Testimonials and reflections on the experience Simulation lab (virtual reality, augmented reality, mixed reality etc) Role playing Use of reflective diaries in clinical placement Reflective writing Final evaluation
Hours	20 h
4th year–gender medicine and relationship with the patient	
Theme	Relationship with the patient
Contents	I Module (1) Review strategies for patient-centered communication (2) Personalized pain assessments (3) Assessment of chronic pain in women II Module (1) The impact of gender on communication and interaction with patients (2) Review strategies for patient-centered communication regardless of gender III Module (1) Ascertaining chronic pain in patients considering the gender difference
Objectives	Deepen knowledge and develop skills on the doctor/patient relationship, in particular in the assessment and therapeutic management of pain
Teaching modalities	Initial autobiography Case discussion Interactive lesson Testimonials and reflections on the experience Simulation lab (virtual reality, augmented reality, mixed reality etc) Role playing Use of reflective diaries in clinical placement Reflective writing Final evaluation
Hours	20 h

## 5. Conclusion

Undergraduate medical students have traditionally been exposed to biophysical-focused curricula with little attention to patients’ needs and expectations. A similar trend has been taking place in clinical research. In addition to a specific focus on biological feedback to new medications and treatments, men represent the predominant population studied.

The predominant role of men in biomedical science has been negatively impacting the delivery of high-quality and personalized medical care to women. Gender bias is equally in place regarding pain assessment and management in the women population. This may drastically impact women’s health and well-being, especially regarding chronic pain and daily living habits. Our purposive literature review and the subsequent exploration of medical schools’ curricula have confirmed this concerning trend. Unfortunately, we could not ascertain relevant practices in this primary educational and medical area as none of the HE institutions we contacted responded to our questionnaire. Furthermore, only 10 universities from the most prestigious academic institutions in the world, as per the Top 10 ranking QS Universities Rankings 2022, were scrutinized. Despite this may constitute a limitation, the research team adopted this strategy due to time constraints and the assumption that world-renowned academic institutions would have posed much more emphasis on gender bias in medical practice and women’s chronic pain management from a non-biomedical perspective.

The proposed educational intervention aims to stimulate a debate within the scientific community and allow the incorporation of a novel approach to limiting the effects of gender bias on future medical practitioners. This innovative approach spreads throughout the undergraduate medical curriculum, ensuring continuity and sustaining an adequate and harmonious development of clinical skills and increased awareness in medical students to manage chronic pain in the female population appropriately. Furthermore, it is worth stressing that gender bias in medical science does not impact negatively only on women’s health as medical science must be as much as possible inclusive, fair, and open to the full spectrum of gender identities.

We recommend this approach in any medical school and curriculum cross-contamination in all other allied health professions university programs in a lifelong interprofessional perspective.

Future studies will include testing the curriculum mentioned above development strategy and exploring its actual and perceived outcomes on the student population.

## Data availability statement

The original contributions presented in the study are included in the article/Supplementary material, further inquiries can be directed to the corresponding authors.

## Author contributions

CM, EDL, CD’A, and AB contributed to the conception and design of the study. CD’A and CM focused on the introduction section. EDL and AB worked on the methodology. All authors focused on the discussion and the curriculum development intervention and contributed to the manuscript revision, and approval of the submitted version.

## Conflict of interest

The authors declare that the research was conducted in the absence of any commercial or financial relationships that could be construed as a potential conflict of interest.

## Publisher’s note

All claims expressed in this article are solely those of the authors and do not necessarily represent those of their affiliated organizations, or those of the publisher, the editors and the reviewers. Any product that may be evaluated in this article, or claim that may be made by its manufacturer, is not guaranteed or endorsed by the publisher.

## Footnotes

^1^By gender bias, we mean the bias generated by stereotypes, i.e., distortions, which occur when gender differences have not been adequately considered. The stereotype (from the Greek “stereos” and “typos,” i.e., “rigid image”) is equivalent to the representation shared by a social group regarding another social group to which characteristics are attributed that derive from approximate generalizations.
