# Supplementary material for: Gender and sex bias in prevention and clinical treatment of women’s chronic pain: hypotheses of a curriculum development
Source: Front Med (Lausanne). 2023 Jul 25;10:1189126. doi: 10.3389/fmed.2023.1189126 (PMC10407111; doi:10.3389/fmed.2023.1189126)
Supplement: Supplementary file 1 [file Table_1.docx]

**Appendix**

**Appendix 1. Questionnaire**

| 1 | Is gender medicine a topic covered in your modules? If yes, could you please name them and share the content and delivery modalities of the teaching materials? |
| --- | --- |
| 2 | Is women's health specifically covered in your modules? If yes, could you please name them and share the content and delivery modalities of the teaching materials? |
| 3 | Is pain assessment/treatment in women specifically covered in your modules? If yes, could you please name them and share the content and delivery modalities of the teaching materials? |
