# Supplementary material for: Gender and sex bias in prevention and clinical treatment of women’s chronic pain: hypotheses of a curriculum development
Source: Front Med (Lausanne). 2023 Jul 25;10:1189126. doi: 10.3389/fmed.2023.1189126 (PMC10407111; doi:10.3389/fmed.2023.1189126)
Supplement: Supplementary file 2 [file Table_2.docx]

Appendix **2. Medical Schools program Humanistic oriented contents – accessible from website**

| **Medical Schools** | **Website contents** | **Programme Humanistic contents** |
| --- | --- | --- |
| ***Asia*** |  |  |
| **NUS Singapore University** | <https://medicine.nus.edu.sg/prospective-students/bachelor-of-medicine-and-bachelor-of-surgery-mbbs/phases-i-and-ii/>  *“Holistic Education, Holistic care begins with a holistic, integrated curriculum...”* | The integrated curriculum is not clear or accessible from the website.  The first 2 years are mostly concerned about physiological aspects of health.  The 3^rd^ year Psychological Medicine is introduced**.**  There are elements of community medicine but they are not presented from a relational or social point of view. |
| **University of Tokyo** | <https://www.u-tokyo.ac.jp/en/whyutokyo/ug_02.html>  *“The Graduate School of Medicine therefore makes every effort to provide an ideal environment for educating specialists in social medicine”.* | Social medicine seems to be the sole humanistic approach proposed to medicine students as a discipline who look at health as a complex phenomenon. |
| **University of South Korea** | <http://www.skkumed.ac.kr/eng/curriculummd.asp>  “*A journey to understand myself, society, human being and human life”.* | The curriculum has modules related to *Humanities Skills*.  In the following years, there are modules on medical humanities: *Patient Experience* and *Doctors and Society.* |
| ***Europe*** |  |  |
| **University of OXFORD** | <https://www.medsci.ox.ac.uk/study/medicine/pre-clinical/structure/pre-clinical-course-structure-description> | The curriculum does not have humanistic contents.  However, there are two modules on *Patient and doctor course* which might have humanistic contents. |
| **Karolinska Institute Sweden** | <https://utbildning.ki.se/programme-syllabus/2LA21>  *“Professional approach is a holistic, humanistic concept that includes knowledge, empathy and self-awareness…”* | The curriculum does not have humanistic or Social sciences contents.  However, on the syllabus are present some skills like “the ability for self-reflection and empathy” and “health-promoting approach with a holistic view of the patient”.  A course on *“The doctor's role in care and society”.*  The content of the teaching is based, among other things, on the competency framework CanMEDS. |
| **Charité - Universitätsmedizin Berlin** | <https://www.charite.de/en/teaching_learning/degree_programs/new_revised_medical_curriculum/> | The curriculum does not have humanistic or Social sciences contents.  However, in their manual it is stated that the teachings are based on a KIT model used to train the students to implement their social and communication skills. This model implies students’ self-reflection and to learn to talk to patients. |
| **University of Leuven** | <https://med.kuleuven.be/en>  *“…an empathic attitude that leaves room for participation and involvement of the patient and his or her relatives.”* | The curriculum does not have humanistic or Social sciences contents.  However, it has been stated that the programme is inspired by a Canadian model and Holistic approach to medicine. |
| ***USA and Canada*** |  |  |
| **Harvard University** | <https://medstudenthandbook.hms.harvard.edu/md-program-objectives> | The curriculum presents some aspects of social sciences.  A Module called Essentials of the Profession (1^st^ year) contains Social Medicine and Foundational communication**.** |
| **Columbia University** | <https://www.vagelos.columbia.edu/education/academic-programs/md-program/curriculum/md-curriculum>  *“We begin with fundamental rapport-building and interviewing techniques and consider the many factors that may impact effective communication and clinical care”.* | The curriculum presents aspects of social sciences called Foundations of Clinical Medicine. Seminars contain the doctor-patient relationship and *narrative medicine.* |
| **Toronto University** | <https://md.calendar.utoronto.ca/education-goals-and-competencies>  <https://md.calendar.utoronto.ca/sites/default/files/PDFs/2021-2022_MD-calendar.pdf> | The curriculum offers an integration of the foundational, social sciences, and humanities to promote the development of *cognitive integration skills*.    They adopt the Can Meds framework which is focused on humanities. |
